# Scutellarin ameliorates pulmonary fibrosis through inhibiting NF-κB/NLRP3-mediated epithelial–mesenchymal transition and inflammation

**DOI:** 10.1038/s41419-020-03178-2

**Published:** 2020-11-13

**Authors:** Ling Peng, Li Wen, Qing-Feng Shi, Feng Gao, Bin Huang, Jie Meng, Cheng-Ping Hu, Chang-Ming Wang

**Affiliations:** 1 Department of Respiratory Medicine (Department of Respiratory and Critical Care Medicine), Key Site of the National Clinical Research Center for Respiratory Disease, Xiangya Hospital, Central South University, Changsha, 410008 P.R. China; 2 Department of Respiratory Medicine, The Fifth Affiliated Hospital of Guilin Medical University, Guilin People’s Hospital, Guilin, 541002 P.R. China

**Keywords:** Diseases, Respiratory tract diseases

## Abstract

Idiopathic pulmonary fibrosis (IPF) is featured with inflammation and extensive lung remodeling caused by overloaded deposition of extracellular matrix. Scutellarin is the major effective ingredient of breviscapine and its anti-inflammation efficacy has been reported before. Nevertheless, the impact of scutellarin on IPF and the downstream molecular mechanism remain unclear. In this study, scutellarin suppressed BLM-induced inflammation via NF-κB/NLRP3 pathway both in vivo and in vitro. BLM significantly elevated p-p65/p65 ratio, IκBα degradation, and levels of NLRP3, caspase-1, caspase-11, ASC, GSDMD^Nterm^, IL-1β, and IL-18, while scutellarin reversed the above alterations except for that of caspase-11. Scutellarin inhibited BLM-induced epithelial–mesenchymal transition (EMT) process in vivo and in vitro. The expression levels of EMT-related markers, including fibronectin, vimentin, N-cadherin, matrix metalloproteinase 2 (MMP-2) and MMP-9, were increased in BLM group, and suppressed by scutellarin. The expression level of E-cadherin showed the opposite changes. However, overexpression of NLRP3 eliminated the anti-inflammation and anti-EMT functions of scutellarin in vitro. In conclusion, scutellarin suppressed inflammation and EMT in BLM-induced pulmonary fibrosis through NF-κB/NLRP3 signaling.

## Introduction

Idiopathic pulmonary fibrosis (IPF) is a chronic, progressive, and irreversible respiratory disease featured with inflammation and extensive lung remodeling caused by overloaded deposition of extracellular matrix^1^. It is reported that the prevalence of IPF is 1.6–1.7 cases in every thousand people^2^. IPF has unclear pathogenesis with the highest morbidity and worst prognosis among the idiopathic interstitial pneumonia family of diseases^3^. Currently, there is no effective treatment for IPF^2,3^. Therefore, the in-depth exploration the pathogenesis and searching other effective drugs have become clinically urgent problems in pulmonary fibrosis.

Despite the pathogenesis of IPF is not fully understood, excessive epithelial–mesenchymal transition (EMT) is suggested to play a crucial role in the development of IPF^2^. Approximately 1/3 fibroblasts of pulmonary fibrosis are identified as epithelial origin. During the EMT process, the E-cadherin in epithelial cells decreases and N-cadherin and α-smooth muscle actin (α-SMA) in mesenchymal cells increase^4^. Myofibroblasts derived from epithelial cells via EMT proliferate rapidly and produce the excessed extracellular matrix^5–7^. Moreover, a previous study indicated that S1PR3 deficiency alleviated radiation-induced pulmonary fibrosis through the regulation of EMT^8^. β-carboline alkaloids could attenuate BLM-induced pulmonary fibrosis in mice through inhibiting EMT^9^. Thus, it is of great significance to explore the regulatory mechanism of EMT in pulmonary fibrosis for finding effective therapeutic drugs.

Except for EMT process, unbalanced inflammation also contributes to the development of IPF^10^. Inflammasomes are large intracellular multiprotein complexes that play a central role in innate immunity^11^. Inflammasomes could respond to the pathogen-associated molecular patterns (PAMPs) and damage-associated molecular patterns (DAMPs)^11^. Nowadays, numerous inflammasomes have been identified and the best characteristic one is NLRP3 inflammasome^12,13^. Once PAMPs or DAMPs are recognized, NF-κB signaling pathway is activated, leading to the upregulated transcription of inflammasome-related components, such as NLRP3, pro-IL-1β, and pro-IL-18. Then the inflammasome-adaptor protein ASC is recruited to NLRP3, then interacts with caspase-1, leading to its activation. Activated caspase-1 can catalyze the maturation of pro-inflammatory cytokines IL-1β and IL-18^14,15^. Recent researches have described the regulatory roles of the NLRP3 inflammasome in various pulmonary diseases including IPF. In a model of bleomycin (BLM)-induced pulmonary fibrosis, Nlrp3−/− mice displayed the reduced neutrophil influx and the reduced IL-1β level in the lung^16^. NLRP3 inflammasome activation and lung fibrosis caused by airborne fine particulate matter were also reported^17^. In addition, NLRP3 was also reported to participate in the regulation of EMT in BLM-induced pulmonary fibrosis^18^. Besides, Huangkui capsule was reported to alleviate renal tubular EMT in diabetic nephropathy via inhibiting NLRP3 inflammasome activation^19^. LFG-500 suppressed EMT in human lung adenocarcinoma cells by inhibiting NLRP3 in inflammatory microenvironment^20^. Thus, inhibiting the EMT process or anti-inflammation through regulating NLRP3 serves as a potential strategy for the treatment of IPF.

Scutellarin (4′,5,6-hydroxyl-flavone-7-glucuronide) is a flavonoid extracted from *Erigeron breviscapus* with multifunctions including anti-oxidation, anti-inflammation, myocardial protection and vascular relaxation^21–23^. Currently, scutellarin is used in the clinical treatment of stroke and myocardial infarction^21,24^. Previous studies reported that scutellarin could suppress NLRP3 inflammasome activation in macrophages and protect mice against bacterial sepsis^22,25^. It was also indicated that scutellarin could exhibit protective effects in retinal hypoxia model by inhibiting NLRP3-mediated inflammatory reaction^23^. Moreover, it inhibited the neuroinflammation via the inhibition of the AKT/NF-κB and p38/JNK pathway in LPS-induced BV-2 microglial cells^26^, and enhanced anti-tumor effects and attenuated the toxicity of bleomycin in H22 ascites tumor-bearing mice^27^. However, there were no studies reported the relation between scutellarin and IPF and the efficacy of scutellarin on IPF is still largely elusive.

In the present study, we reported that scutellarin reduced inflammation and EMT process in BLM-induced pulmonary fibrosis. Our study reported that scutellarin ameliorated pulmonary fibrosis through modulating NF-κB/NLRP3-mediated EMT and inflammatory response. This is the first study demonstrating the effects of scutellarin on IPF. Our study provided its potential molecular mechanism and a novel candidate for IPF treatment.

## Material and methods

### Establishment of pulmonary fibrosis model and animals grouping

Eighty 8-week-old male BALB/c mice with weight about 20–30 g, were purchased from SJA Laboratory Animal company (Hunan, China) and housed at the animal research facility of Xiangya Hospital, Central South University (Changsha, Hunan, China). Mice were acclimatized to the laboratory condition and provided free access to ad libitum food and water. The mice were accommodated for 7 days before experiments and housed in an environment of 50 ± 10% relative humidity, 24 ± 2 °C, and a 12-h light/dark cycle. Animals were randomly divided into five groups: control group, BLM-induced pulmonary fibrosis group, BLM-induced pulmonary fibrosis with three different concentrations of scutellarin groups (30, 60, and 90 mg/kg). Pulmonary fibrosis model was induced by intratracheal instillation of bleomycin (MedChem Express, Princeton, NJ, USA) at the concentration of 5.0 mg/kg under anesthesia by isoflurane. Mice in the control group were received intracheal instillation of the same amount of saline solution. In other groups, mice were established with pulmonary fibrosis model and treated with scutellarin (MedChem Express, Princeton, NJ, USA) by intragastric administration once per day for 14 or 28 days. After 14 or 28 days, mice were sacrificed, lung tissues and blood were then collected for further measurements. The accessing process was conducted by an assessor blind to treatment allocation. All experiments were conducted in accordance to the ethical guidelines and all protocols were approved by the Animal Care and Use Committee of the Xiangya Hospital, Central South University (Changsha, Hunan, China).

### H&E staining

Isolated lung tissues were fixed using 4% paraformaldehyde at room temperature for 24 h and then embedded in paraffin, and were cut into 4 μm sections using a rotary microtome (Leica, Mannheim, Germany). The dried slices were soaked in xylene, dewaxed for 10 min, rehydrated, then performed hematoxylin rinse for 5 min and washed, followed by differentiation with 1% hydrochloric acid alcohol. Then slides were stained with eosin for 30 s, dehydrated with gradient alcohol, soaked in xylene three times. Finally, mounted slides with neutral gum and analyzed under an optical microscope (DP73; Olympus Corporation, Tokyo, Japan). The process of taking images was conducted by an assessor blind to treatment allocation.

### Masson’s trichrome staining

The formation of collagen fibers was examined with Masson’s trichrome staining as manufacture’s instruction. Briefly, slides were stained in Weigert’s iron hematoxylin working solution for 10 min, then washed in water and followed by Biebrich scarlet-acid fuchsin solution for 15 min. Slides were stained with aniline blue solution after color differentiated. Finally, slides were dehydrated with ethanol, cleared in xylene and mounted with resinous mounting medium and analyzed under an optical microscope lastly (DP73; Olympus Corporation, Tokyo, Japan). The process of taking images was conducted by an assessor blind to treatment allocation.

### Immunohistochemistry (IHC)

For IHC staining, slides were stained with antibodies against NLRP3 (1:200, Abcam, USA), E-cadherin (1:100, Cell Signaling Technology, USA) and N-cadherin (1:200, Santa Cruz, USA) before hematoxylin co-staining. After incubation overnight at 4 °C and rinsing 2 × 5 min TBS 0.025% Triton, incubated it with the secondary antibody diluted in buffer according to the manufacturer’s instructions. The staining of NLRP3, E-cadherin, and N-cadherin was analyzed under an optical microscope (DP73; Olympus Corporation, Tokyo, Japan). The process of taking images was conducted by an assessor blind to treatment allocation.

### Cell culture and transfection

The human lung epithelial A549 cell line and the rat lung epithelial RLE-6TN cell line were purchased from American Type Culture Collection (ATCC, Manassas, VA, USA). All the cell lines included in this study have been authenticated by STR profiling and tested for mycoplasma contamination. A549 cells were grown in Dulbecco’s modified Eagle’s medium (DMEM) supplemented with 25 mM sodium bicarbonate, 10 mM HEPES, 100 U/mL penicillin, 100 μg/mL streptomycin and 10% fetal bovine serum (FBS). RLE-6TN cells were cultured with F12 medium containing 2 mM l-glutamine, 10 μg/mL bovine pituitary extract, 5 μg/mL insulin, 1.25 μg/mL transferrin, 2.5 ng/mL insulin-like growth factor, 2.5 ng/mL epidermal growth factor, 100 U/mL penicillin, 100 μg/mL streptomycin and 10% FBS. Cells were cultured at 37 °C in humidified atmosphere with 5% CO_2_. Reagents for cell culture were all purchased from Life Technologies (Gibco, Grand Island, NY, USA).

For cell treatment, cells were divided into five groups: control group, BLM-induced pulmonary fibrosis group, BLM-induced pulmonary fibrosis with 0.1, 0.2, or 0.4 mM scutellarin groups. Cells were pretreated with 0.1, 0.2, or 0.4 mM of scutellarin for 2 h, then 50 μM (RLE-6TN) or 100 μM (A549) of BLM was added to induce pulmonary fibrosis for 24 or 48 h. For cell transfection experiment, pcDNA3.1-NLRP3 overexpression vector (OE-NLRP3) and pcDNA3.1-NC vector (OE-NC) were designed and synthesized from Shanghai GenePharma Co., Ltd and were transfected into cells using Lipofectamine^®^ 3000 reagent (Invitrogen).

### MTT assay

3-(4,5-Dimethylthiazol-2-yl)-2,5-diphenyl tetrazoliumbromide (MTT) assay was used to assess the proliferation of cells. Briefly, cells were seeded in 96-well plates at 2 × 10^4^ cells/well. Then, cells were grouped and treated with indicated treatment. 24 or 48 h after treatment, the medium was removed and 20 μL of MTT dye solution (5 mg/mL in PBS buffer, Sigma) was added per well and incubate at 37 °C for 4 h. Then supernatant was decanted and replaced with 150 μL DMSO. After 15 min incubation with gentle shaking at 37 °C, the absorbance was measured at a wavelength of 490 nm using microtiter plate reader.

### Immunofluorescence assay

Cells were fixed with 4% paraformaldehyde, washed with PBS, and permeabilized with 0.1% Triton X-100 for 5 min. Samples were then blocked for 1 h with 5% bovine serum albumin in PBS. Then cells were incubated with a goat anti-NLRP3 (Abcam, USA) primary antibody for overnight at 4 °C. Specific secondary antibody conjugated with GFP fluorochrome (Invitrogen, Carlsbad, CA) was incubated for 1 h at room temperature. After PBS washes, slides were mounted. Images were captured using an optical microscope (DP73, Olympus Corporation, Tokyo, Japan). The process of taking images was conducted by an assessor blind to treatment allocation.

### ELISA assay

ELISA was employed to determine the concentration of IL-1β and IL-18 in serum and supernatant fluid of cultured cells. ELISA kits were purchased from Abcam and measurement was carried out according to the manufacturer’s protocols.

### Modulation of MMPs activity

A EnzChek™ Gelatinase/Collagenase Assay Kit (E12055, Thermo Fisher Scientific, USA) was used to detect MMPs activity in cells as described before^28^ and according to the manufacturer’s protocols.

### RNA extraction and reverse transcription-quantitative PCR

Total RNA was extracted from tissues and cells with TRIzol reagent (Invitrogen) according to the manufacturer’s instruction. Samples were reversely transcribed to complementary DNA (cDNA) using the PrimeScript® RT reagent Kit with gDNA Eraser (Takara, Dalian, China). The gene expression was measured by qPCR with SYBR Premix EX Taq Kit (Takara, Dalian, China) and Applied Biosystems 7500 Fast RealTime qPCR machine (ThermoFisher, USA). GAPDH was used as internal control. Relative mRNA expression levels were quantified with 2^−ΔΔCt^ method. Primers used for RT-qPCR were synthesized by Sangon Biotech Co., Ltd.

### Western blotting

Proteins were extracted from tissues or cells using RIPA buffer (Beyotime) and concentrations were determined by a bicinchoninic acid assay Kit (Thermo Fisher Scientific, Inc.). Then equal aliquots of proteins were separated on 8–10% SDS-PAGE. Following electrophoresis, the proteins were transferred onto PVDF membranes (EMD Millipore, Billerica, MA, USA), and blocked with 5% BSA for 1 h at room temperature. The membranes were then incubated overnight at 4 °C with the primary antibodies against α-SMA, collagen I, p-p65, p65, IκBα, NLRP3, caspase-1, caspase-11, ASC, GSDMD^Nterm^, IL-1β, IL-18, fibronectin, vimentin, E-cadherin, N-cadherin, MMP-2/9, snail, and GAPDH. GAPDH was used as the internal control. The membranes were then washed with TBST buffer and incubated with HRP-conjugated secondary antibodies for 1 h at room temperature. All antibodies were purchased from Abcam, Santa Cruz, or Cell Signaling Technology. Protein bands were then visualized using the enhanced chemiluminescence detection system. The intensity of the bands was quantified using ImageJ software tools.

### Statistical analysis

All experiments were performed in at least three biological replicates, and each biological replicate contained three technical replicates. All statistical analyses were performed by Graphpad Prim 5 (GraphPad Software, La Jolla, CA, USA). Data were presented as mean ± standard deviation (SD). All the data meet the assumption of normal distribution. For comparisons between two groups, Student’s *t*-test (two tailed) was employed. Comparisons among ≥3 groups were conducted using one-way analysis of variance (ANOVA) followed by Tukey’s test. *P* < 0.05 was considered statistically significant.

## Results

### Scutellarin ameliorated lung damages in BLM-induced pulmonary fibrosis in vivo

**Fig. 1:**
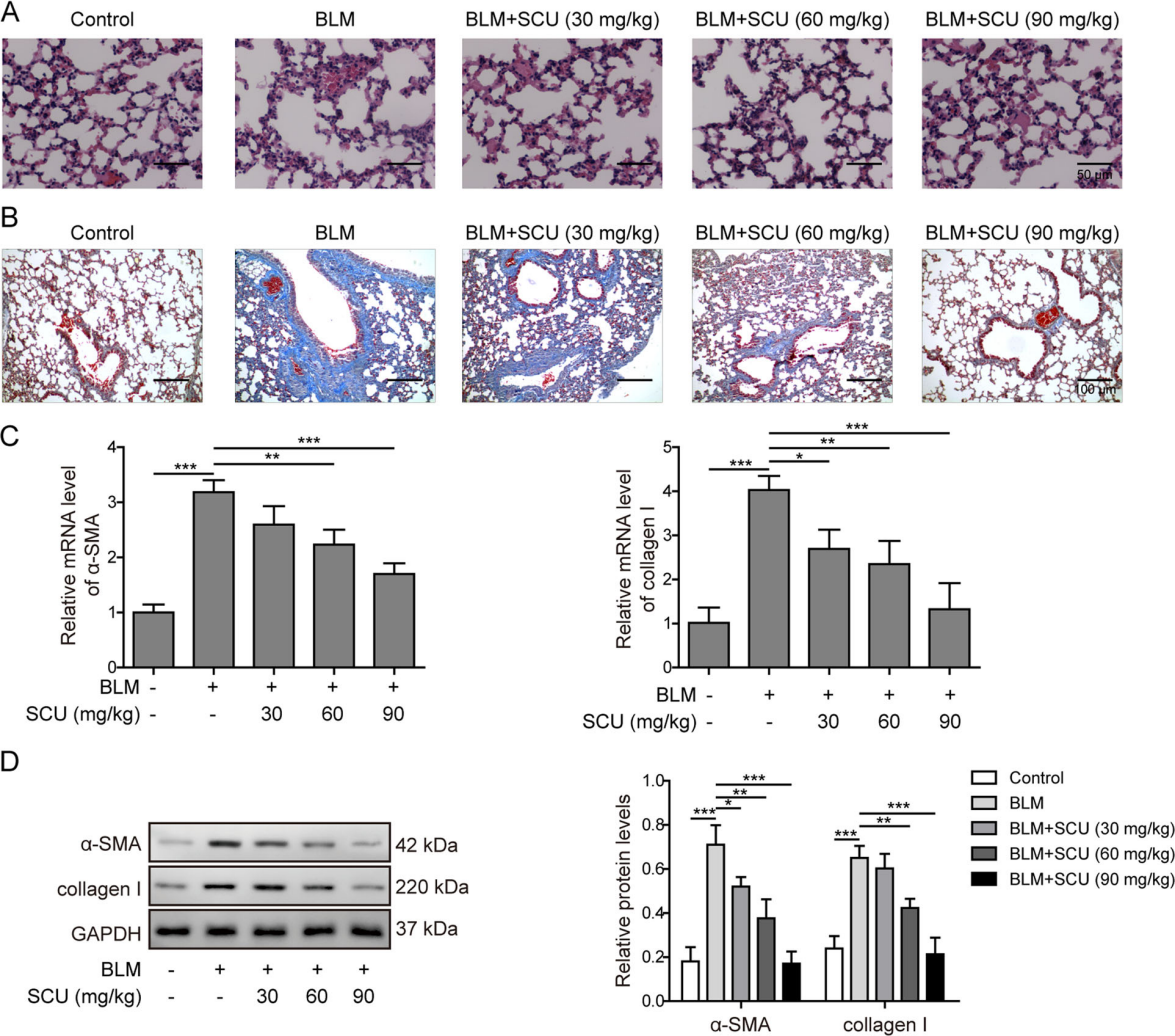
Scutellarin attenuated BLM-induced collagen deposition in pulmonary fibrosis mice model. **A** Lung tissues structures visualized with H&E staining. Scale bar: 50 μm. **B** Collagen deposition in lung tissues detected with Masson’s trichrome staining. Scale bar: 100 μm. **C** The mRNA levels of α-SMA and collagen I measured with RT-qPCR. **D** Protein levels of α-SMA and collagen I measured with western blotting. *P < 0.05, **P < 0.01, and **P < 0.001.

To investigate the effects of scutellarin in fibrosis, mice were administrated with BLM to induce pulmonary fibrosis. Compared with the control, pulmonary fibrosis mice demonstrated the thickened alveolar septa and increased mononuclear infiltration throughout the lung parenchyma. After the scutellarin treatment, the fibrosis symptoms were significantly reduced (Fig. 1A). Fibrillar collagen deposition, an indicator of pulmonary fibrosis, was confirmed with Masson’s trichrome staining. Masson’s staining showed the marked blue stained collagen was observed in lung tissues of fibrosis mice. Meanwhile, scutellarin dose-dependently ameliorated the collagen deposition in lung tissues (Fig. 1B). Then, we measured the mRNA levels of α-SMA and collagen I, biomarkers of fibrosis, with RT-qPCR. Data revealed that BLM induced the expression levels of α-SMA and collagen I, which was reversed by scutellarin in a dose-dependent manner (Fig. 1C). Furthermore, western blotting demonstrated similar changes in protein levels, indicating that scutellarin could efficiently improve BLM-induced pulmonary fibrosis in mice (Fig. 1D). Taken together, these results indicated that scutellarin could ameliorate lung damages in BLM-induced pulmonary fibrosis in vivo.

### Scutellarin suppressed BLM-induced inflammation via NF-κB/NLRP3 pathway in vivo

**Fig. 2:**
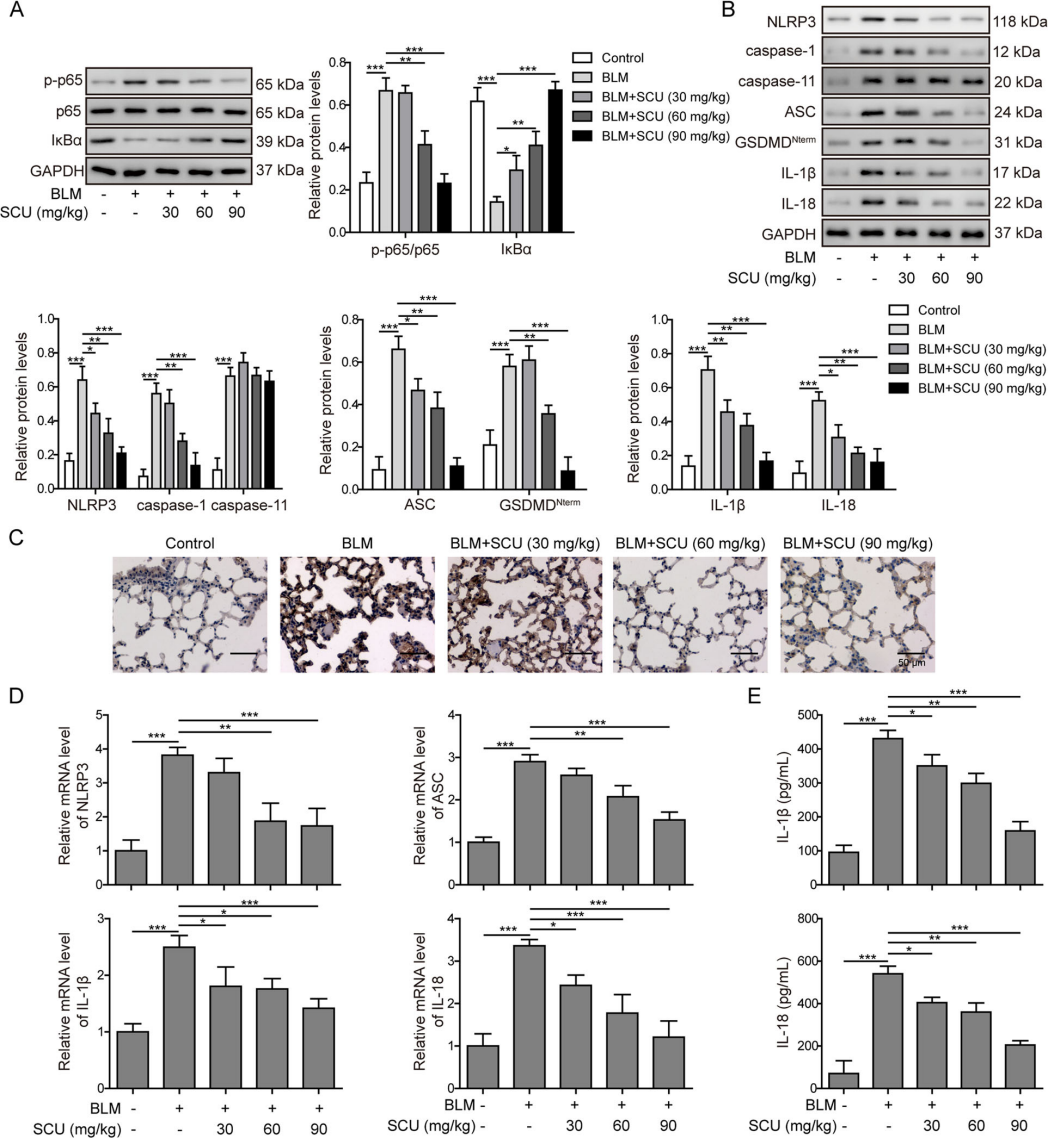
Scutellarin alleviated NF-κB/NLRP3 signaling pathway in BLM-induced pulmonary fibrosis. **A** Protein levels of phosphorylated p65 (p-p65), total p65 and IκBα measured with western blotting. **B** Protein levels of NLRP3, caspase-1, caspase-11, ASC, GSDMD^Nterm^, IL-1β, and IL-18 measured with western blotting. **C** NLRP3 expression visualized with immunohistochemistry staining. Scale bar: 50 μm. **D** The mRNA levels of NLRP3, ASC, IL-1β, and IL-18 measured with RT-qPCR. **E** The concentration of IL-1β and IL-18 in serum detected with ELISA. *P < 0.05, **P < 0.01, and **P < 0.001.

Previous studies demonstrated that NF-κB/NLRP3 signaling pathway promoted liver fibrosis in mice^29^. Thus, we wanted to explore if the activation of NF-κB/NLRP3 signaling was involved in pulmonary fibrosis and if scutellarin regulated this pathway. Western blotting demonstrated that BLM significantly elevated the ratio of phosphorylated p65 to total p65 (p-p65/p65) and reduced the expression level of IκBα, indicating that NF-κB signaling pathway was activated in BLM-induced pulmonary fibrosis. However, scutellarin treatment down-regulated the ratio of p-65/p65 and induced higher expression of IκBα in a dose-dependent way (Fig. 2A), indicating that scutellarin suppressed BLM-induced activation of NF-κB signaling pathway. Besides, the expression levels of NLRP3, caspase-1, caspase-11, ASC, GSDMD^Nterm^, IL-1β, and IL-18 were elevated in BLM group, which could also be compensated by scutellarin treatment except for caspase-11 (Fig. 2B). Immunohistochemistry was employed to visualize the in-situ expression of NLRP3 in lung tissues. As shown in Fig. 2C, BLM increased the expression of NLRP3, and scutellarin dramatically reversed the elevation of NLRP3 (Fig. 2C). RT-qPCR demonstrated that mRNA levels of NLRP3, ASC, IL-1β, and IL-18 were also upregulated in BLM group, but scutellarin reduced them in a dose-dependent manner (Fig. 2D). Consistently, ELISA results demonstrated that BLM-induced IL-1β and IL-18 production in serum was dose-dependently suppressed by scutellarin (Fig. 2E). Taken together, BLM activated NF-κB/NLRP3 signaling pathway, resulting in the increased production of IL-1β and IL-18 in mice.

### Scutellarin reduced BLM-induced EMT process in vivo

**Fig. 3:**
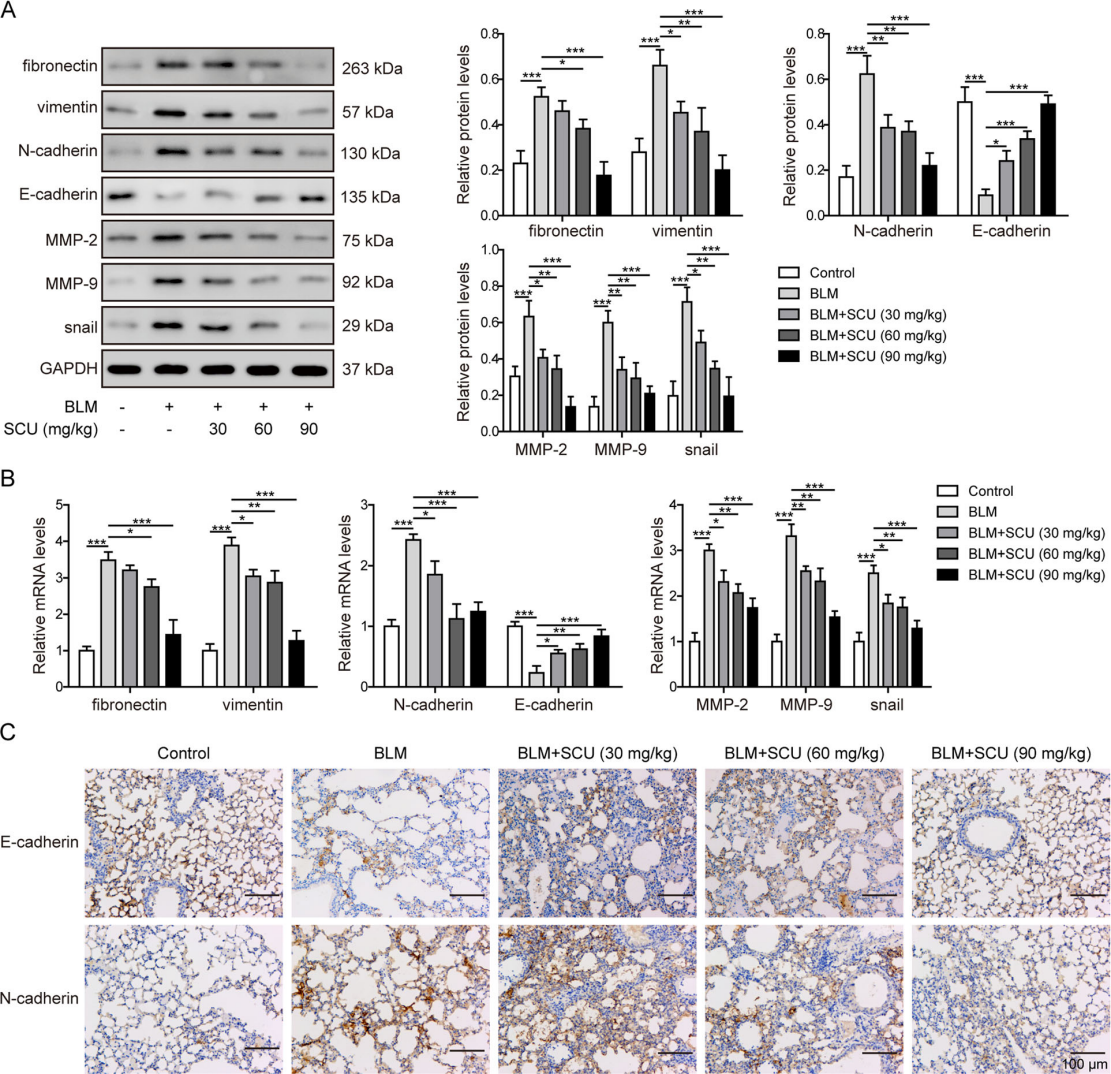
Scutellarin reduced BLM-induced EMT in pulmonary fibrosis. **A** Protein levels of fibronectin, vimentin, E-cadherin, N-cadherin, MMP-2, MMP-9, and snail measured with western blotting. **B** The mRNA levels of fibronectin, vimentin, E-cadherin, N-cadherin, MMP-2, MMP-9, and snail measured with RT-qPCR. **C** The expression levels of E-cadherin and N-cadherin detected by IHC. *P < 0.05, **P < 0.01 and **P < 0.001.

Previous studies demonstrated that EMT was related with fibrosis such as kidney fibrosis^30^ and hepatic fibrosis^29^. EMT mediated by NF-κB/NLRP3 signaling pathway participated in the development of pulmonary fibrosis^18,20^. Here, we investigated the expression levels of proteins in lung tissues about EMT on day 28. Western blotting revealed that fibronectin, vimentin, N-cadherin, MMP-2, MMP-9, and snail were increased in BLM group, which were reversed by scutellarin treatment (Fig. 3A). Oppositely, scutellarin maintained the level of cell adhesive protein E-cadherin, which was significantly reduced in BLM-induced pulmonary fibrosis mice (Fig. 3A). Consistently, mRNA levels of fibronectin, vimentin, N-cadherin, MMP-2, MMP-9, and snail were also increased, and E-cadherin level was reduced in BLM group. Meanwhile, scutellarin dose-dependently reversed these changes above (Fig. 3B). Besides, the expressions of E-cadherin and N-cadherin were further detected by IHC. Figure 3C showed that N-cadherin was increased while E-cadherin was decreased in BLM group, which were reversed by scutellarin treatment. Similar results were observed on day 14 (Supplementary Fig. 1). Fibronectin, vimentin, N-cadherin, MMP-2, MMP-9, and snail expression levels were increased in BLM group, which were reversed by scutellarin treatment both on protein and mRNA levels on day 14 (Supplementary Fig. 1A, B). Oppositely, scutellarin maintained the level of E-cadherin, which was significantly reduced in BLM-induced pulmonary fibrosis mice on day 14 (Supplementary Fig. 1A, B). Moreover, IHC showed that N-cadherin was increased while E-cadherin was decreased in BLM group, which were reversed by scutellarin treatment on day 14 (Supplementary Fig. 1C). Taken together, these results indicated that the extracellular deposition of collagen was mediated by EMT and scutellarin could efficiently suppress BLM-induced EMT in vivo.

### Scutellarin inhibited BLM-induced pulmonary fibrosis in vitro

To investigate the mechanism by which scutellarin suppressed BLM-induced pulmonary fibrosis, we firstly established pulmonary fibrosis in vitro model by stimulating A549 and RLE-6TN cells with BLM for 24 or 48 h. We determined the concentrations of scutellarin with MTT assay and data revealed that concentrations above 0.8 mM demonstrated cytotoxicity in both cells (Supplementary Fig. 2A). Thus, in the following studies, scutellarin was used at the dose of 0.1, 0.2, and 0.4 mM. As shown in Supplementary Fig. 2B, BLM caused severe viability loss and scutellarin dose-dependently rescued the viability in both cell lines. It’s noting that cells in 48 h treatment of BLM demonstrated lower viability than that of 24 h group (Supplementary Fig. 2B). RT-qPCR revealed that scutellarin suppressed BLM-induced expression levels of α-SMA and collagen I (Supplementary Fig. 2C, D). Western blotting data indicated that the protein levels of these proteins were also decreased by scutellarin (Supplementary Fig. 2E), which was in accordance with the results obtained in animal model. In a word, scutellarin delivered similar anti-pulmonary fibrosis efficacy in vitro.

### Scutellarin suppressed BLM-induced inflammation via NF-κB/NLRP3 pathway in vitro

**Fig. 4:**
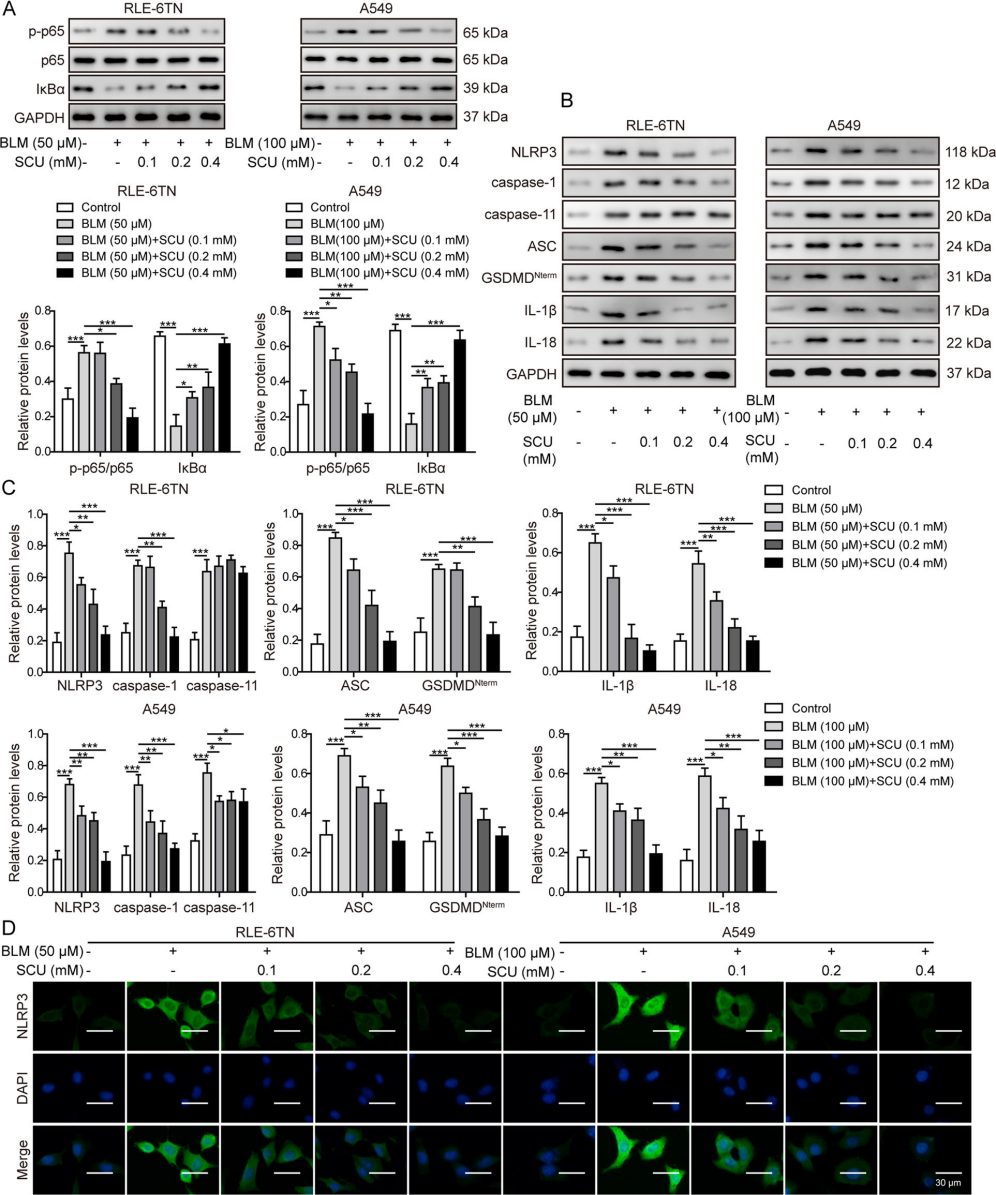
Scutellarin inhibited the activation of NF-κB/NLRP3 signaling pathway induced by BLM in vitro. **A** Protein levels of phosphorylated p65 (p-p65), total p65 and IκBα measured with western blotting. **B** Protein levels of NLRP3, caspase-1, caspase-11, ASC, GSDMD^Nterm^, IL-1β, and IL-18 measured with western blotting. **C** Quantification of western blotting in B. **D** NLRP3 expression visualized with immunofluorescence staining. Scale bar: 30 μm. *P < 0.05, **P < 0.01, and **P < 0.001.

Next, we confirmed the activation of NF-κB/NLRP3 pathway in BLM-stimulated cell model. Western blotting revealed that p-p65/p65, NLRP3, caspase-1, caspase-11, ASC, GSDMD^Nterm^, IL-1β, and IL-18 were upregulated by BLM, while IκBα was reduced. Scutellarin reversed the above effects (Fig. 4A–C). Immunofluorescence staining showed that NLRP3 expression was elevated in BLM group, and scutellarin reversed this increase (Fig. 4D). Consistently, RT-qPCR confirmed the increase of mRNA levels of NLRP3, ASC, IL-1β, and IL-18 by BLM, and scutellarin inhibited these effects (Supplementary Fig. 3A–D). ELISA results demonstrated that the concentrations of IL-1β and IL-18 in supernatant were significantly increased as well after BLM treatment. Nevertheless, the scutellarin treatment dose-dependently reduced the production of inflammatory cytokines IL-1β and IL-18 (Supplementary Fig. 3E, F), suggesting that the scutellarin could deliver anti-inflammatory function in pulmonary fibrosis in vitro and NF-κB/NLRP3 pathway might participate in this regulation process.

### Scutellarin inhibited BLM-induced EMT process in vitro

**Fig. 5:**
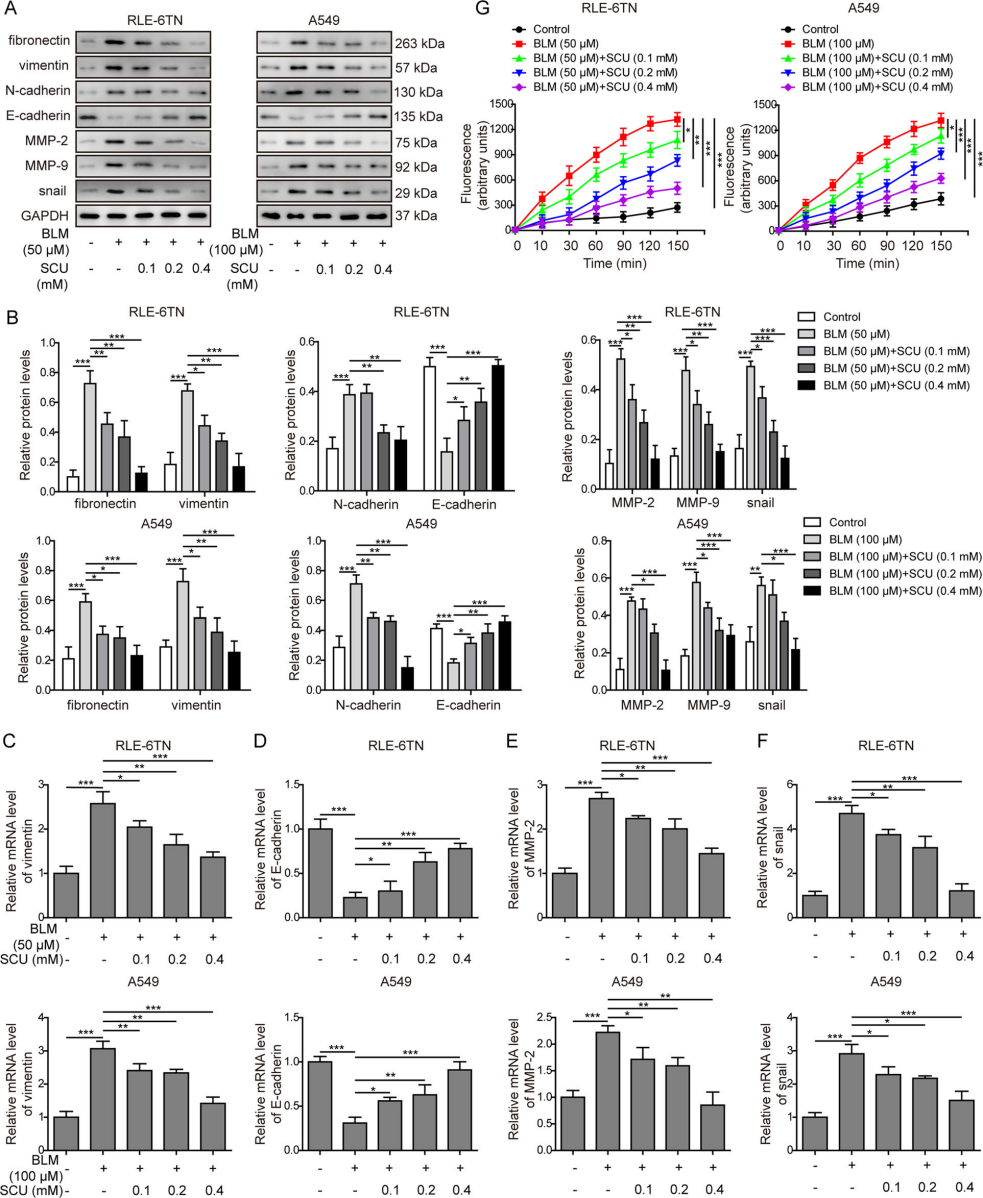
Scutellarin inhibited BLM-induced EMT in vitro. **A** Protein levels of fibronectin, vimentin, E-cadherin, N-cadherin, MMP-2, MMP-9, and snail measured with western blotting. **B** Quantification of western blotting in **A**. **C** The mRNA level of vimentin measured with RT-qPCR. **D** The mRNA level of E-cadherin measured with RT-qPCR. **E** The mRNA level of MMP-2 measured with RT-qPCR. **F** The mRNA level of snail measured with RT-qPCR. **G** MMP activity was measured via a EnzChek™ Gelatinase/Collagenase Assay Kit. *P < 0.05, **P < 0.01, and **P < 0.001.

Furthermore, we investigated the mechanism by which scutellarin regulated EMT in BLM-induced pulmonary fibrosis in vitro. Consistent with what observed in vivo, both RLE-6TN and A549 cells exposed to BLM expressed higher protein levels of fibronectin, vimentin, N-cadherin, MMP-2, MMP-9, and snail, as well as less E-cadherin (Fig. 5A, B). The mRNA levels of vimentin, E-cadherin, MMP-2, and snail presented the similar changes (Fig. 5C–F). In addition, scutellarin could dose-dependently reverse these changes (Fig. 5A–F). Moreover, MMP activity was measured via a commercial kit. Results in Fig. 5G showed that BLM enhanced the MMP activity while scutellarin could dose-dependently reverse these changes. Therefore, scutellarin could reverse BLM-induced EMT process in vitro.

### NLRP3 overexpression reversed effects of scutellarin on inflammation

**Fig. 6:**
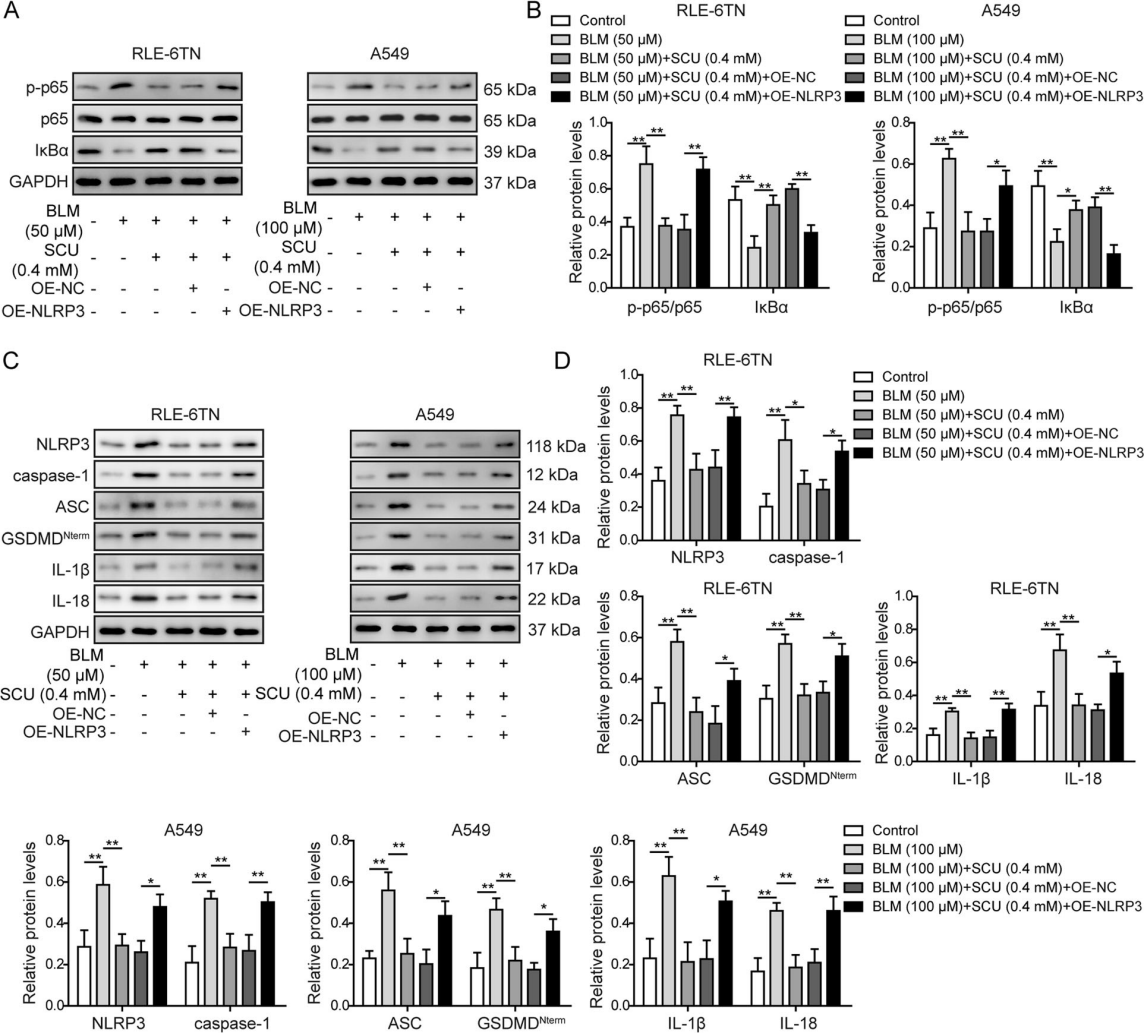
NLRP3 abrogated down-regulation of proteins in NF-κB/NLRP3 pathway mediated by scutellarin. **A** Protein levels of phosphorylated p65 (p-p65), total p65 and IκBα measured with western blotting. **B** Quantification of western blotting in **A**. **C** Protein levels of NLRP3, caspase-1, ASC, GSDMD^Nterm^, IL-1β, and IL-18 measured with western blotting. **D** Quantification of western blot in **C**. *P < 0.05 and **P < 0.01.

To further confirm the role of NLRP3 in scutellarin-mediated anti-inflammation efficacy, we overexpressed NLRP3 in both cell lines. The success of overexpression was validated with western blotting, as the protein level of NLRP3 was increased significantly compared to cells transfected with pcDNA3.1-NC (Fig. 6C, D). The inhibition on the ratio of p-p65/p65, and the protein expression of caspase-1, ASC, GSDMD^Nterm^, IL-1β and IL-18 by 0.4 mM of scutellarin was abrogated by the overexpression of NLRP3 (Fig. 6A–D). On the other hand, IκBα protein was upregulated by the overexpression of NLRP3 (Fig. 6A, B). Consistently, the effects of scutellarin on mRNA levels of NLRP3, ASC, IL-1β, and IL-18 were abrogated by the overexpression of NLRP3 (Supplementary Fig. 4A–D). What’s more, the production of IL-1β and IL-18 in NLRP3-overexpressed RLE-6TN and A549 was increased again (Supplementary Fig. 4E, F). Taken together, scutellarin delivered anti-inflammatory efficacy in pulmonary fibrosis through inhibiting BLM-induced NF-κB/NLRP3 signaling, and overexpression of NLRP3 partly eliminated the function of scutellarin.

### NLRP3 overexpression reversed effects of scutellarin on EMT and extracellular matrix deposition

**Fig. 7:**
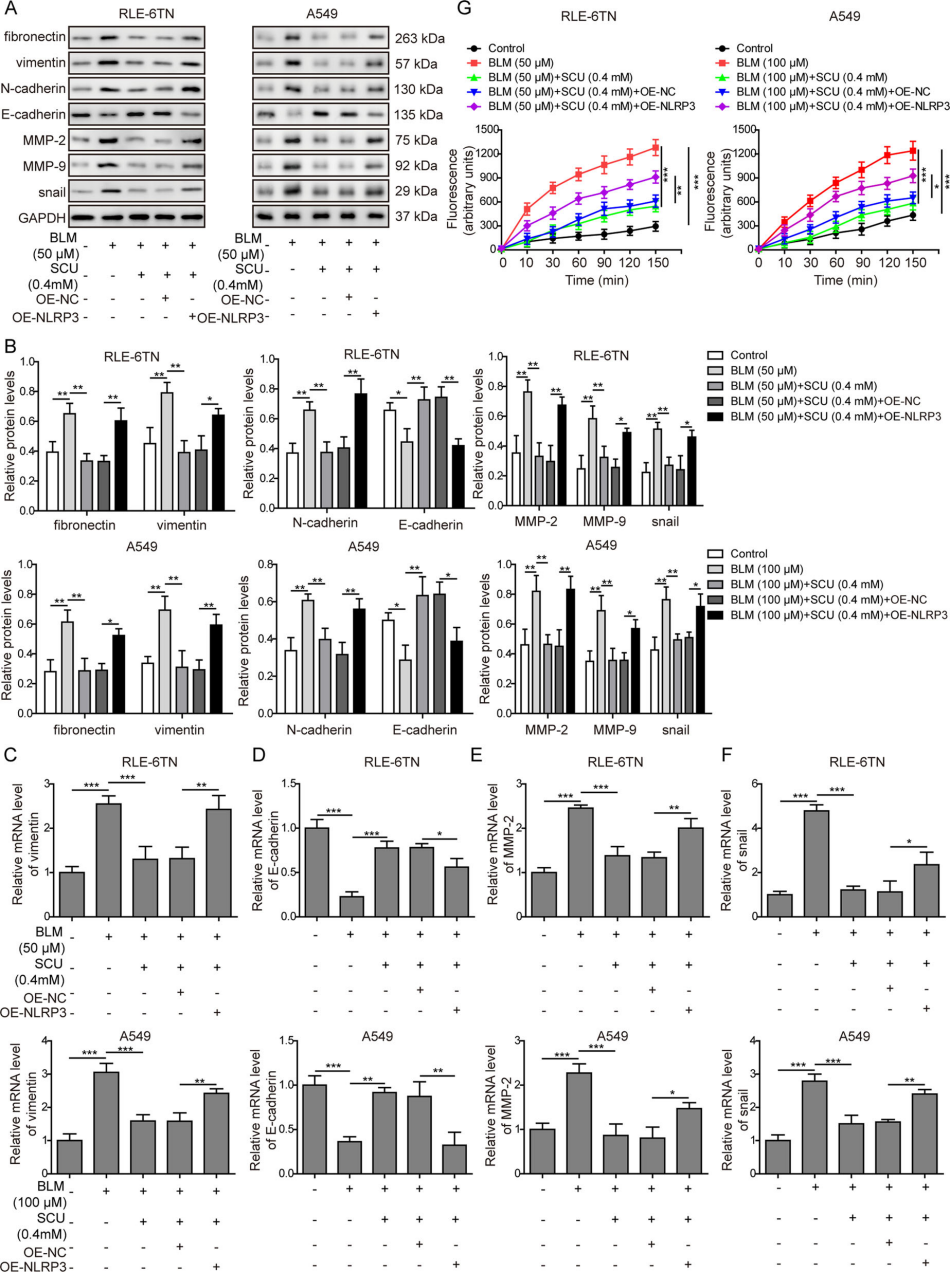
Overexpression of NLRP3 abrogated the EMT suppression efficacy of scutellarin in vitro. **A** Protein levels of fibronectin, vimentin, E-cadherin, N-cadherin, MMP-2, MMP-9, and snail measured with western blotting. **B** Quantification of western blotting in **A**. **C** The mRNA level of vimentin measured with RT-qPCR. **D** The mRNA level of E-cadherin measured with RT-qPCR. **E** The mRNA level of MMP-2 measured with RT-qPCR. **F** The mRNA level of snail measured with RT-qPCR. **G** MMP activity was measured via a EnzChek™ Gelatinase/Collagenase Assay Kit. *P < 0.05, **P < 0.01, and **P < 0.001.

**Fig. 8:**
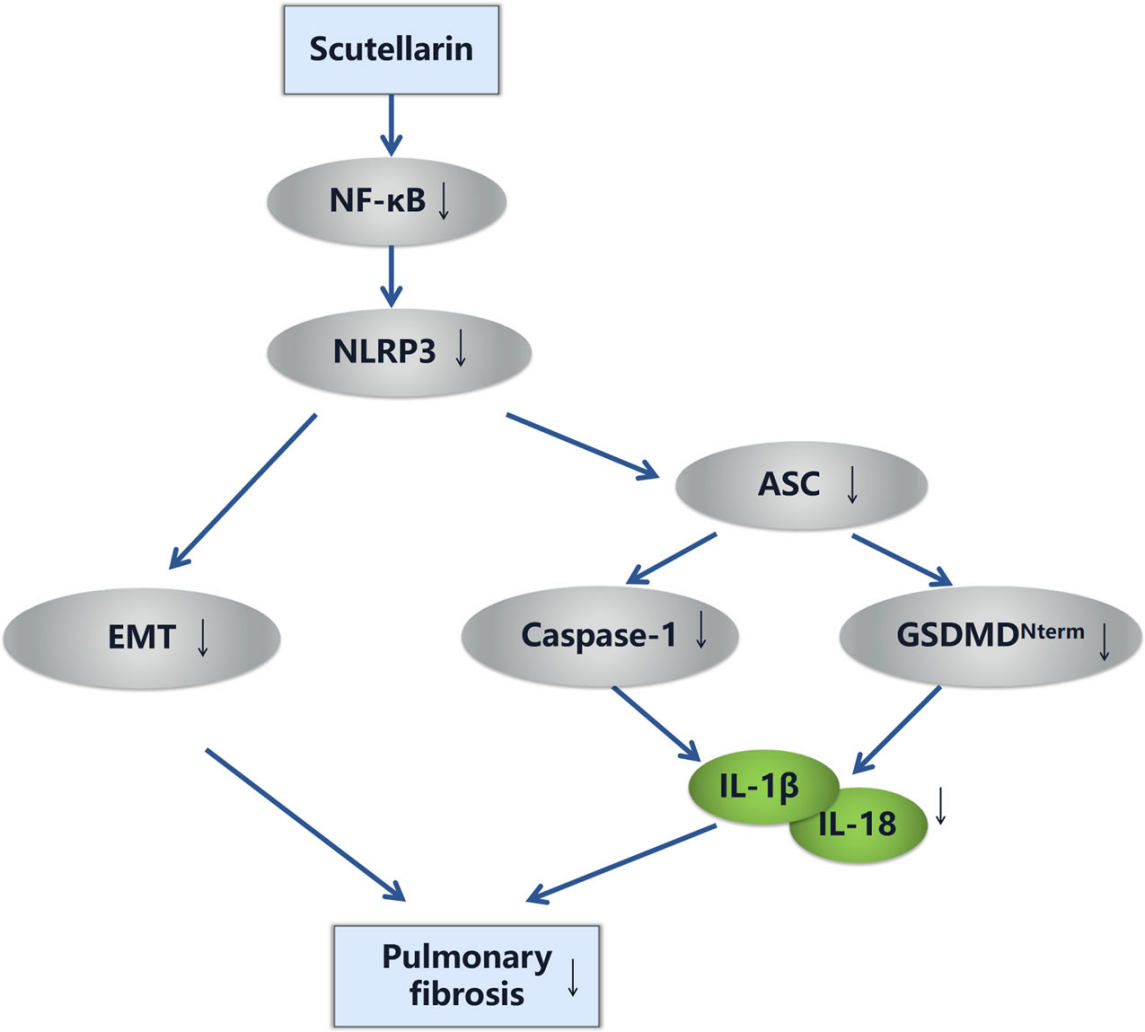
Roadmap of this article. Scutellarin suppressed inflammation and EMT in BLM-induced pulmonary fibrosis through NF-κB/NLRP3 signaling pathway.

Finally, we hoped to verify whether scutellarin inhibited BLM-induced EMT through NLRP3, we found that NLRP3 overexpression abrogated the inhibition on fibronectin, vimentin, N-cadherin, MMP-2, MMP-9 and snail, and reversed the promotion on E-cadherin in protein and mRNA levels by scutellarin (Fig. 7A–F). Moreover, MMP activity was measured and results in Fig. 7G showed that NLRP3 overexpression abrogated the inhibition of MMP activity caused by scutellarin. What’s more, overexpression of NLRP3 significantly increased the expression of α-SMA and collagen I, compared to scutellarin treatment group (Supplementary Fig. 5A, B), indicating that scutellarin inhibited EMT in BLM-induced pulmonary fibrosis through suppressing the expression of NLRP3, and manually elevating the NLRP3 level eliminated the anti-EMT efficacy of scutellarin (Fig. 8).

## Discussion

Pulmonary fibrosis is a chronic, progressive lung disease, featured with rapid and lethal respiratory failure and high mortality^31^. IPF, the most common form of pulmonary fibrosis, affects 8–14% of patients each year^32–34^. Scutellarin is the major effective ingredient of breviscapine and its anti-inflammation efficacy has been reported before^35^. However, the effect of scutellarin on pulmonary fibrosis is not fully reported yet, and the underlying mechanism needs further investigation. Here in vitro and in vivo pulmonary fibrosis models were performed, and we reported that scutellarin could efficiently inhibit BLM-induced inflammation response and EMT process. Further studies revealed that scutellarin regulated inflammation response through inhibiting NF-κB pathway, resulting in downregulation of NLRP3 and the production of IL-1β and IL-18. Our study unraveled a novel mechanism for scutellarin in treating pulmonary fibrosis.

We demonstrated that scutellarin ameliorated BLM-induced pulmonary fibrosis histopathologically and through measuring α-SMA and collagen I both in vivo and in vitro. Nie et al. demonstrated that scutellarin enhanced the anti-tumor effect of BLM and reduced BLM-induced pulmonary fibrosis^27^, which was consistent with our discovery. Scutellarin also plays regulatory role in fibrosis of other organ. For instance, scutellarin suppressed platelet aggregation and fibrogenesis in mouse^36^. Besides, scutellarin showed anti-fibrosis effect via inhibition of EMT on isoprenaline-induced myocardial fibrosis in rats^37^. However, this is the first systematic study to prove and investigate the regulatory role of scutellarin in BLM-induced pulmonary fibrosis.

Pulmonary fibrosis is a chronic inflammation disease in lung tissues. Inflammation response plays a complex role in the development of fibrosis. Balanced collagen synthesis and degradation are disrupted in pulmonary fibrosis, as more extracellular matrix accumulation in tissues^38^. Increased collagen fragments can be pro-inflammatory^39^. Pulmonary fibrosis can also be alleviated through inhibiting inflammation. For instance, NLRP3/IL-1β/TGF-β signal axis was associated with silicosis pulmonary fibrosis^40^, and aucubin alleviated BLM-induced pulmonary fibrosis and reduced the intrapulmonary collagen disposition and inflammatory injury induced by BLM in mice^25^. What’s more, neutralization of IL-18 by IL-18 binding protein ameliorated bleomycin-induced pulmonary fibrosis via inhibition of EMT^41^. Here, we revealed that scutellarin could reverse the upregulation of caspase-1 instead of caspse-11 induced by BLM. NLRP3 inflammasomes are reported to activate caspase-1, which leads to the maturation of IL-1β and IL-18 and induction of pyroptosis^42^. On the other hand, pyroptosis is also induced by the activation of murine caspase-11 of non-canonical inflammasomes^43^. Though, a recent study reported that caspase-11 non-canonical inflammasome activation could also induce the activation of the NLRP3/ASC/caspase-1 pathway, this newly identified “non-canonical” NLRP3 inflammasome activation is still distinguished from the “canonical” NLRP3 inflammasome activation^44^. Thus, we inferred that scutellarin alleviated BLM-induced inflammation via suppressing canonical NLRP3 inflammasome activation, and we for the first time discovered that scutellarin could suppress inflammation in pulmonary fibrosis by inhibiting NLRP3. Consistent with our findings, scutellarin suppressed NLRP3 inflammasome activation in macrophages^35^. Meanwhile, scutellarin also protected against myocardial ischemia–reperfusion injury by suppressing NLRP3 inflammasome activation^45^.

Studies have shown that EMT is an important factor in the development of pulmonary fibrosis^18^. Immunohistochemistry staining of lung tissues from IPF patients demonstrated extending E-cadherin expression into the basal cells, where the expression levels of vimentin and N-cadherin were increased^46^, suggesting that epithelium cells were in the process of transition to mesenchymal cells. In BLM-induced pulmonary fibrosis mice, inhibiting EMT with TGF-β inhibitor sufficiently ameliorated pulmonary fibrosis^47^. Also, sulforaphane attenuated pulmonary fibrosis by inhibiting EMT^48^. Besides, calpain inhibition attenuated bleomycin-induced pulmonary fibrosis via switching the development of EMT^49^. Moreover, activation of NF-κB was found to activate the expression of potent EMT inducers, like snail and zeb, suggesting a close link between fibrosis, inflammation, and EMT^50,51^. NLRP3 inflammasome activation could promote the EMT process during the fibrosis^52^. In the present study, we also investigated whether scutellarin regulated EMT process through NF-κB/NLRP3 pathway in BLM-induced pulmonary fibrosis both in vitro and in vivo. We revealed that scutellarin regulated EMT-related gene expression such as fibronectin, vimentin, E-cadherin, N-cadherin, MMP-2, MMP-9, and snail in vivo and in vitro. We further showed that scutellarin inhibited the EMT through regulating NLRP3, which, as far as we known, was the first study that reported the regulatory role of scutellarin on EMT in BLM-induced pulmonary fibrosis. MMP activities were also measured and results indicated that BLM enhanced the MMP activity while scutellarin dose-dependently reversed these changes and NLRP3 overexpression abrogated the inhibition of MMP activity caused by scutellarin. This study also suggested that targeting EMT served as a potential strategy for pulmonary fibrosis treatment. It was well known that NF-κB used to serving as the upstream of NLRP3^53^. However, overexpression of NLRP3 can also lead to the increase of IL-1β and IL-18, and many literatures reported that IL-1β feedback loop could promote the activation of NF-κB signaling pathway^54–59^. Therefore, in our results, overexpression of NLRP3 caused the activation of NF-κB signaling pathway, although NF-κB served as the upstream of NLRP3.

Recently, Gabasa et al. reported that EMT did not contribute directly to the myofibroblast population, and may contribute to the stiff fibrotic microenvironment through their own stiffness but not their collagen expression^60^. This is in stark contrast to our finding. However, research on EMT in pulmonary fibrosis has been still reported in the lots of literatures recently^61–66^. There is no conclusion in this field. We hope more evidence will emerge and help us to further elucidate whether EMT process is of much significance in pulmonary fibrosis. In addition, many literatures used TGF-β1 to induce in vitro lung fibrosis models^62,67^. Bleomycin was also identified to be used to induce lung fibrosis and inflammation in vitro^18,68,69^. Therefore, in our study we initially used bleomycin to establish the in vitro model of lung fibrosis and induced the synthesis of collagen I. For the mechanism of pulmonary fibrosis, EMT transformation of epithelial cells into fibroblasts and myofibroblasts leads to the proliferation of these cells, and the accumulation of a large amount of extracellular matrix. Therefore, after EMT transformation, epithelial cells may already have the characteristics of myofibroblasts to some extent, and collagen I is produced. Furthermore, other studies also showed that collagen I in lung fibrosis was from epithelial cells which undergone EMT transformation^70–73^. Nowadays novel approaches such as cell linage tracing and single cell RNA seq (scRNA-seq) analyses do help us understand fibrosis better. For example, scRNA-seq analyses identified loss of normal epithelial cell identities and unique contributions of epithelial cells to the pathogenesis of IPF^74^. Also, scRNA-seq identified type I interferon (IFN) response signature as a potential source of diagnostic and prognostic markers of renal fibrosis^75^. Moreover, scRNA-seq analyses also classified and defined six mesenchymal cell types in normal lung and seven in fibrotic lung^76^. Therefore, we completely agree with the reviewer that variety of methods would strengthen our manuscript. We will delve deeply into other methods and seriously consider it in our future studies. In this study, we have carried out some innovations in ideas: First, this is the first systematic report that scutellarin relieves pulmonary fibrosis. Then, we are the first to report that scutellarin inhibits the activation of NLRP3 in pulmonary fibrosis. Up till now, there are only two reports about scutellarin inhibiting NLRP3, one in sepsis^22^ and the other in myocardial ischemia reperfusion^45^. Besides, this is the first report that scutellarin inhibits EMT in pulmonary fibrosis.

To conclude, in the present study, we reported that a natural-derived scutellarin significantly ameliorated inflammation response and EMT process through NF-κB/NLRP3 signal pathway in BLM-induced pulmonary fibrosis. Thus, we unraveled the underlying mechanism of scutellarin in ameliorating pulmonary fibrosis, providing a novel candidate for the treatment of pulmonary fibrosis.

## Supplementary information

Supplementary Figure legends

Supplementary Table 1

supplementary fig.1

supplementary fig.2

supplementary fig.3

supplementary fig.4

supplementary fig.5
