# Supplementary material for: Scutellarin ameliorates pulmonary fibrosis through inhibiting NF-κB/NLRP3-mediated epithelial–mesenchymal transition and inflammation
Source: Cell Death Dis. 2020 Nov 13;11(11):978. doi: 10.1038/s41419-020-03178-2 (PMC7666141; doi:10.1038/s41419-020-03178-2)
Supplement: Supplementary file 1 — Supplementary Figure legends [file 41419_2020_3178_MOESM1_ESM.docx]

**Supplementary Figure Legends**

**Supplementary figure 1. Scutellarin reduced BLM-induced EMT in pulmonary fibrosis on day 14.**

(A) Protein levels of fibronectin, vimentin, E-cadherin, N-cadherin, MMP-2, MMP-9 and snail measured with western blotting on day 14. (B) The mRNA levels of fibronectin, vimentin, E-cadherin, N-cadherin, MMP-2, MMP-9 and snail measured with RT-qPCR on day 14. (C) The expression levels of E-cadherin and N-cadherin detected by IHC on day 14. *P<0.05, **P<0.01 and **P<0.001.

**Supplementary figure 2.** **Scutellarin reduced BLM-induced pulmonary fibrosis *in vitro*.**

(A) Cell viability of RLE-6TN and A549 cells measured with MTT assay. RLE-6TN and A549 cells were treated with scutellarin at different doses for 24 h or 48 h. (B) Cell viability of RLE-6TN and A549 cells measured with MTT assay. Cells were treated with BLM with/without scutellarin at different doses for 24 h or 48 h. (C) The mRNA levels of α-SMA measured with RT-qPCR. (D) The mRNA levels of collagen I measured with RT-qPCR. (E) The protein levels of α-SMA and collagen I measured with western blotting. *P<0.05, **P<0.01 and **P<0.001.

**Supplementary figure 3.** **Scutellarin reversed the up-regulation of the expressions of markers in NF-κB/NLRP3 pathway induced by BLM *in vitro*.**

(A) The mRNA level of NLRP3 measured with RT-qPCR. (B) The mRNA level of ASC measured with RT-qPCR. (C) The mRNA level of IL-1β measured with RT-qPCR. (D) The mRNA level of IL-18 measured with RT-qPCR. (E) The concentration of IL-1β in supernatant measured with ELISA. (F) The concentration of IL-18 in supernatant measured with ELISA. *P<0.05, **P<0.01 and **P<0.001.

**Supplementary figure 4.** **NLRP3 abrogated down-regulation of markers in NF-κB/NLRP3 pathway mediated by scutellarin.**

(A) The mRNA level of NLRP3 measured with RT-qPCR. (B) The mRNA level of ASC measured with RT-qPCR. (C) The mRNA level of IL-1β measured with RT-qPCR. (D) The mRNA level of IL-18 measured with RT-qPCR. (E) The concentration of IL-1β in supernatant measured with ELISA. (F) The concentration of IL-18 in supernatant measured with ELISA. *P<0.05, **P<0.01 and **P<0.001.

**Supplementary Figure 5.** **NLRP3 abrogated the collagen deposition suppression efficacy of scutellarin *in vitro*.**

(A) Protein levels of α-SMA and collagen I measured with western blotting. (B) Quantification of western blotting in A. *P<0.05 and **P<0.01.
