# Supplementary material for: Scutellarin ameliorates pulmonary fibrosis through inhibiting NF-κB/NLRP3-mediated epithelial–mesenchymal transition and inflammation
Source: Cell Death Dis. 2020 Nov 13;11(11):978. doi: 10.1038/s41419-020-03178-2 (PMC7666141; doi:10.1038/s41419-020-03178-2)
Supplement: Supplementary file 2 — Supplementary Table 1 [file 41419_2020_3178_MOESM2_ESM.docx]

| Antibodies | Company | Product number | Species reactivity | Species; Polyclonal or monoclonal | Molecular  weight (kDa) |
| --- | --- | --- | --- | --- | --- |
| α-SMA | CST | 19245 | H,M,R | Rabbit mAb | 42 |
| Collagen I | CST | 91144 | H,M,R | Rabbit mAb | 220 |
| p-p65 | CST | 3039 | H,M,R | Rabbit pAb | 65 |
| p65 | CST | 8242 | H,M,R,Hm,Mk,Dg | Rabbit mAb | 65 |
| IκBα | CST | 9247 | H,M,R | Mouse mAb | 39 |
| NLRP3 | Abcam | ab263899 | H,M,R | Rabbit mAb | 118 |
| NLRP3 | Abcam | ab4207 | H,R | Goat pAb | 118 |
| NLRP3 | Abcam | ab214185 | H,M,R | Rabbit pAb | 118 |
| Caspase-1 | Abcam | ab179515 | H,M,R | Rabbit mAb | 12 |
| Caspase-11 | Santa cruz | sc-374615 | H,M,R | Mouse mAb | 20 |
| ASC | Santa cruz | sc-514414 | H,M,R | Mouse mAb | 24 |
| GSDMD^Nterm^ | Abcam | ab215203 | H,M,R | Rabbit mAb | 31 |
| IL-1β | Santa cruz | sc-52012 | H,M,R | Mouse mAb | 17 |
| IL-18 | Abcam | ab191860 | R,M | Rabbit pAb | 22 |
| IL-18 | CST | 54943 | H | Rabbit mAb | 22 |
| Fibronectin | Abcam | ab45688 | H,M,R | Rabbit mAb | 263 |
| Vimentin | Santa cruz | sc-6260 | H,M,R | Mouse mAb | 57 |
| E-cadherin | CST | 14472 | H,M,R | Mouse mAb | 135 |
| N-cadherin | Santa cruz | sc-59987 | H,M,R | Mouse mAb | 130 |
| MMP-2 | Abcam | ab86607 | H,M,R | Mouse mAb | 75 |
| MMP-9 | Abcam | ab58803 | H,M,R | Mouse mAb | 92 |
| Snail | CST | 3879 | H,M,R,Mk | Rabbit mAb | 29 |
| GAPDH | CST | 5174 | H,M,R,Mk | Rabbit mAb | 37 |

Supplementary Table 1: The detailed information of antibodies used.

H, human; M, mouse; R, rat; Hm, hamster; Mk, monkey; Dg, Dog; CST, Cell Signaling Technology;
