# Supplementary figures and images for: Scutellarin ameliorates pulmonary fibrosis through inhibiting NF-κB/NLRP3-mediated epithelial–mesenchymal transition and inflammation
Source: Cell Death Dis. 2020 Nov 13;11(11):978. doi: 10.1038/s41419-020-03178-2 (PMC7666141; doi:10.1038/s41419-020-03178-2)

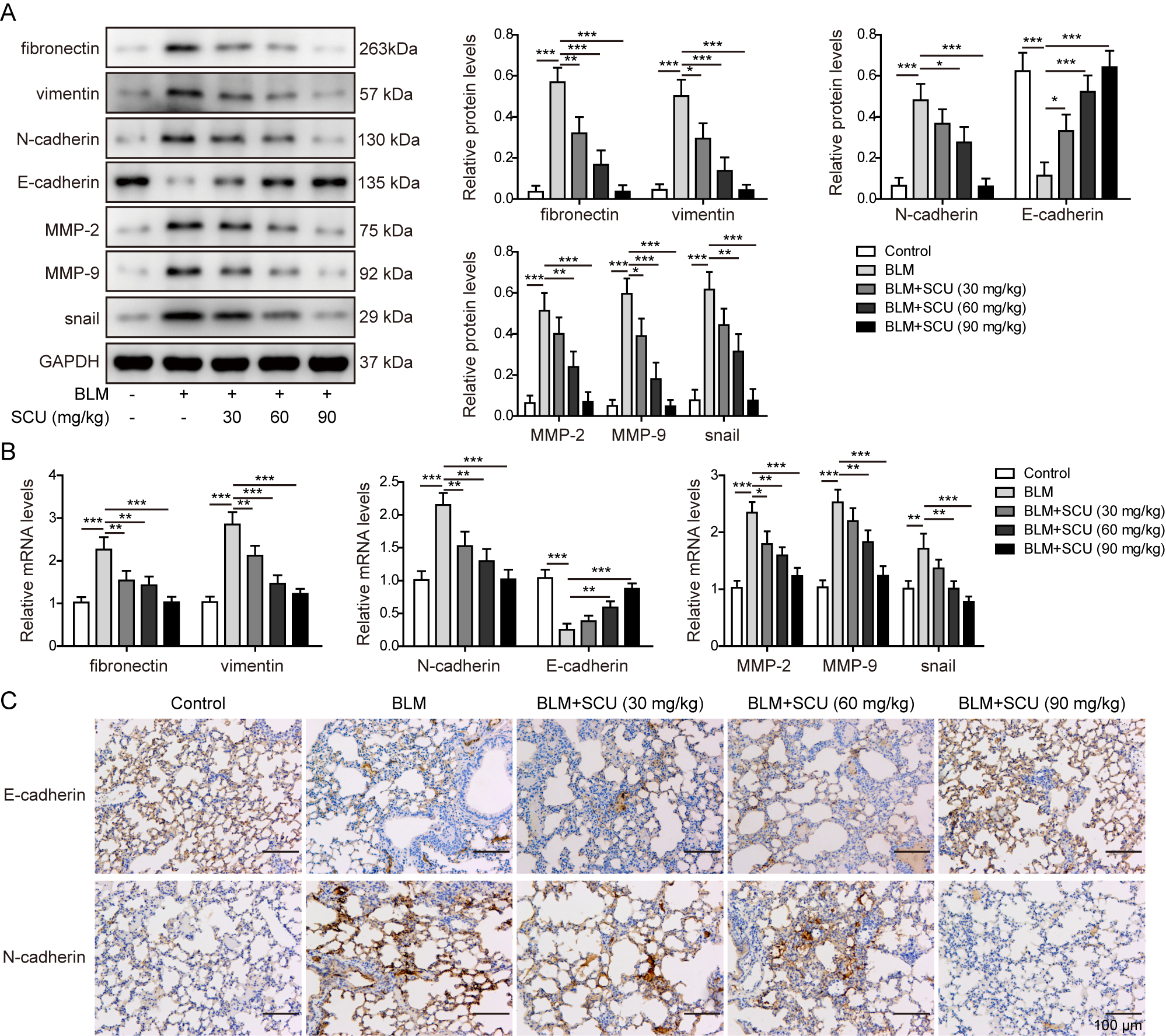

Supplement: Supplementary file 3 — supplementary fig.1 [file 41419_2020_3178_MOESM3_ESM.tif]

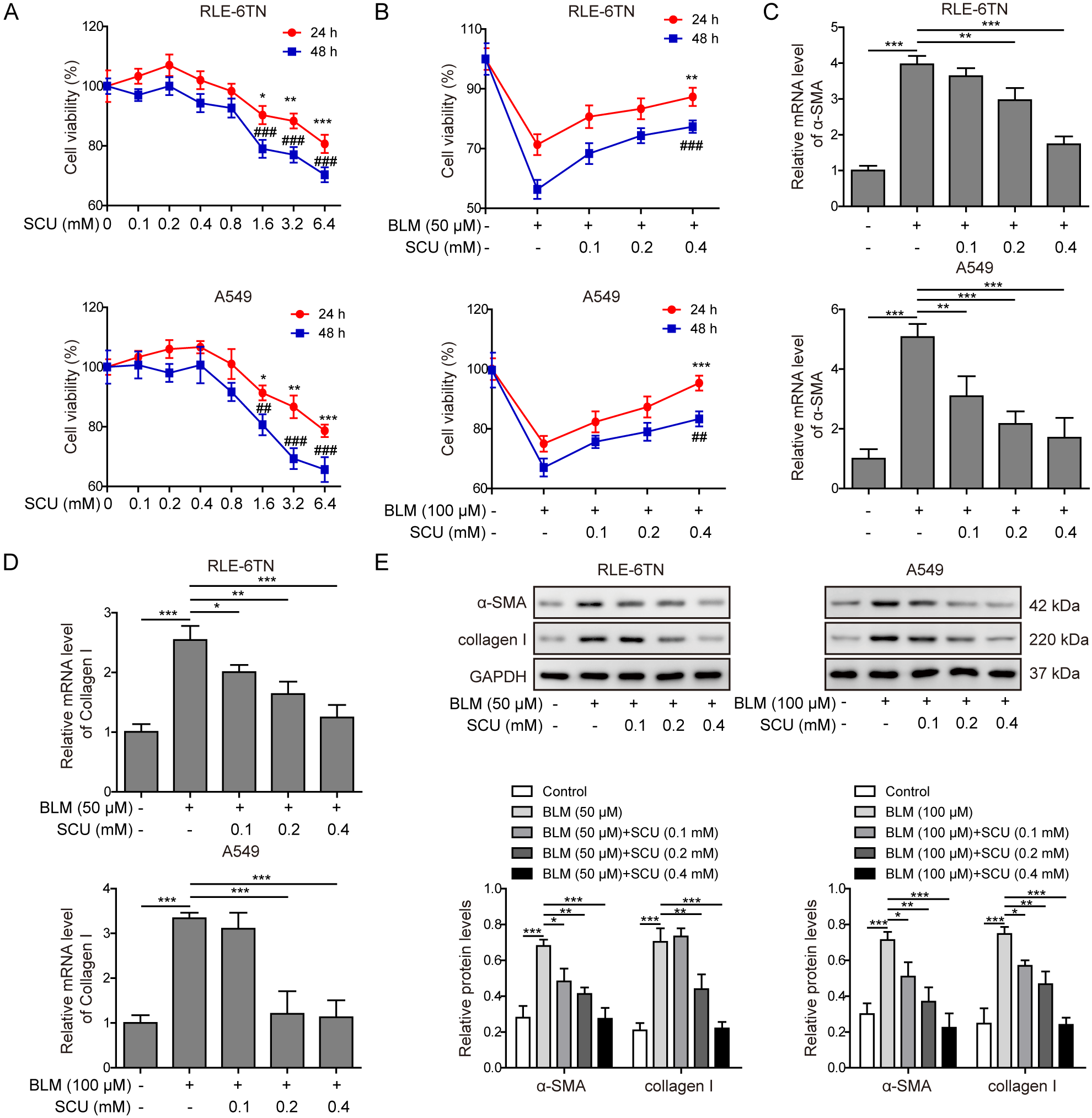

Supplement: Supplementary file 4 — supplementary fig.2 [file 41419_2020_3178_MOESM4_ESM.tif]

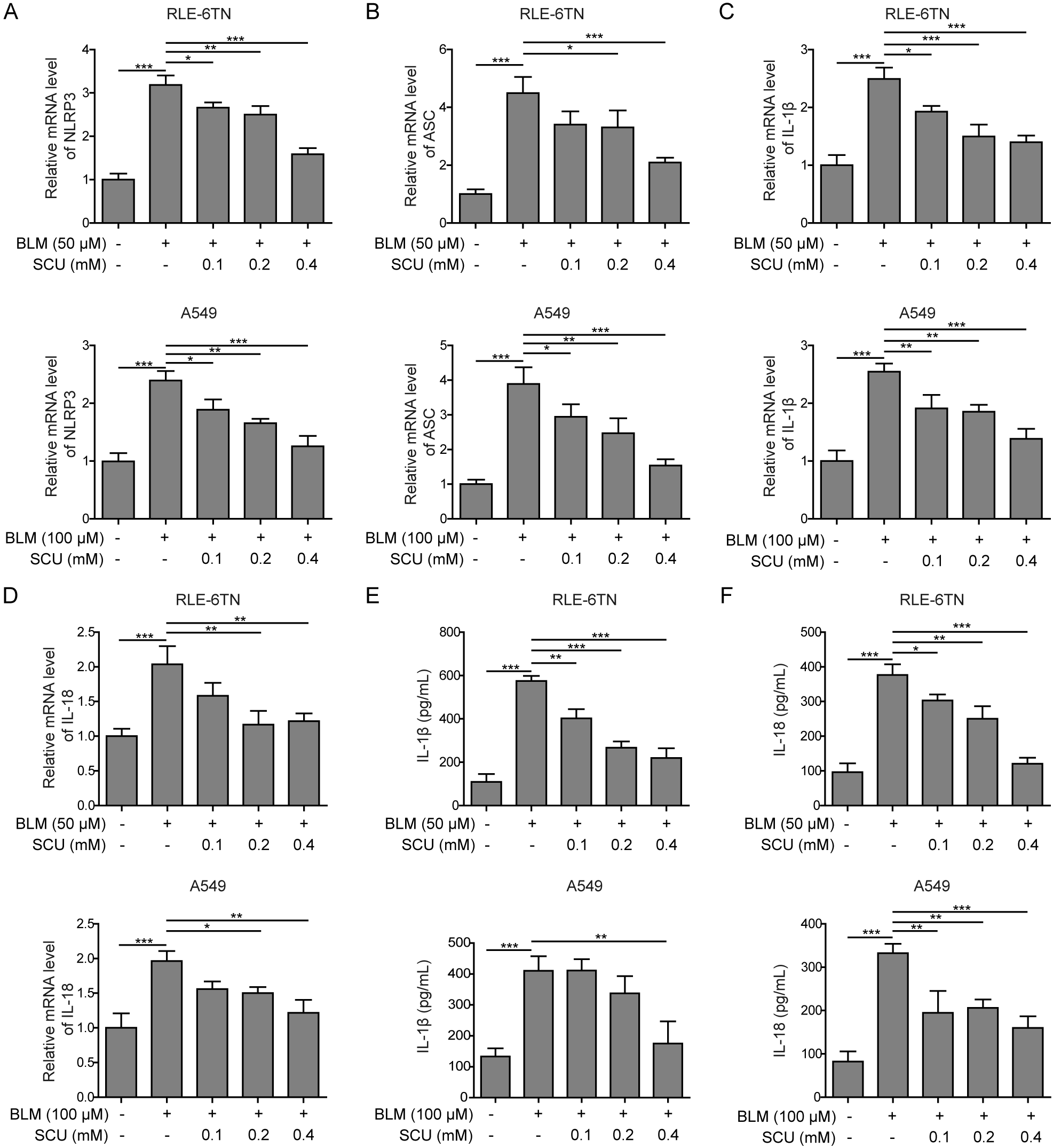

Supplement: Supplementary file 5 — supplementary fig.3 [file 41419_2020_3178_MOESM5_ESM.tif]

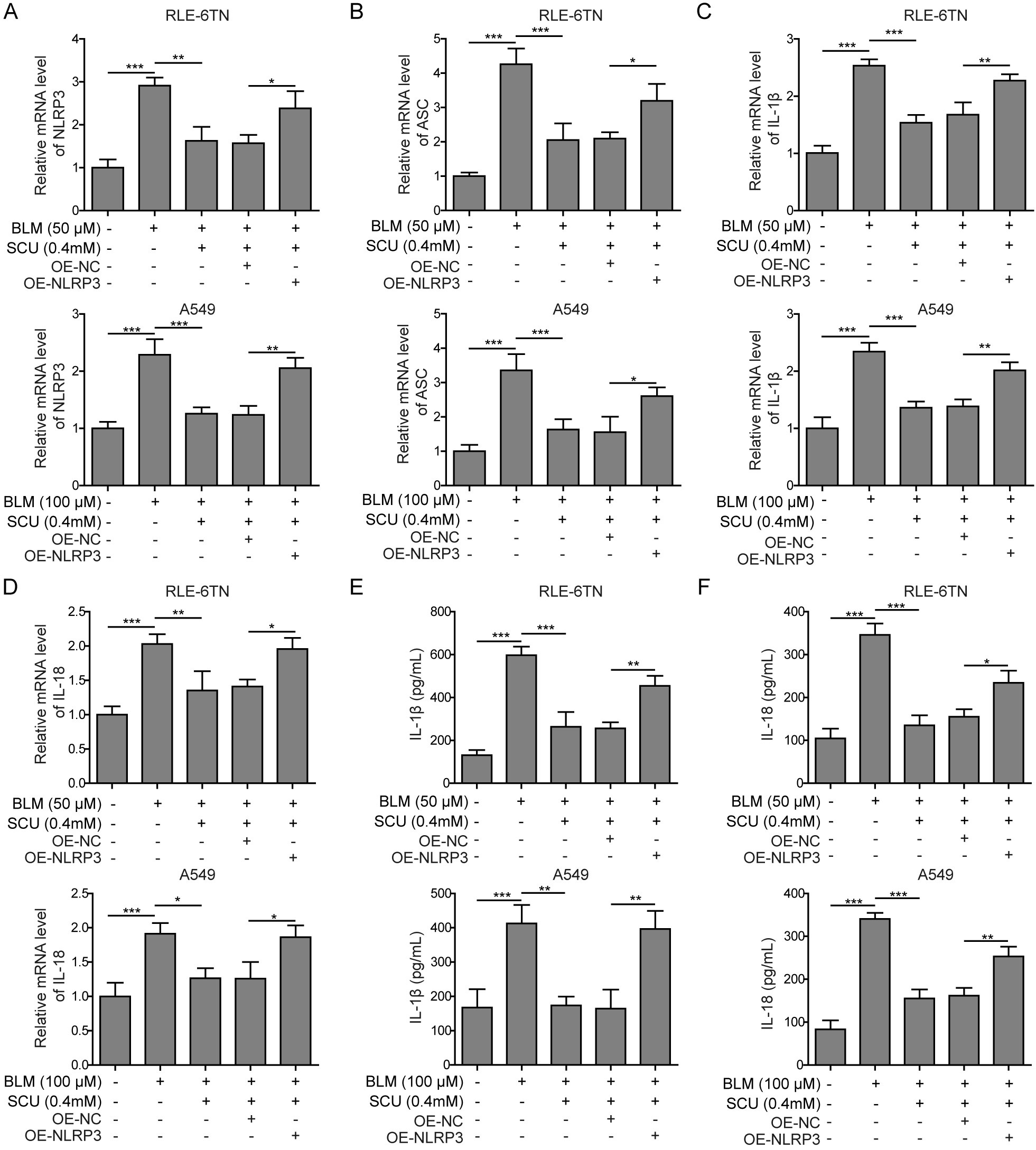

Supplement: Supplementary file 6 — supplementary fig.4 [file 41419_2020_3178_MOESM6_ESM.tif]

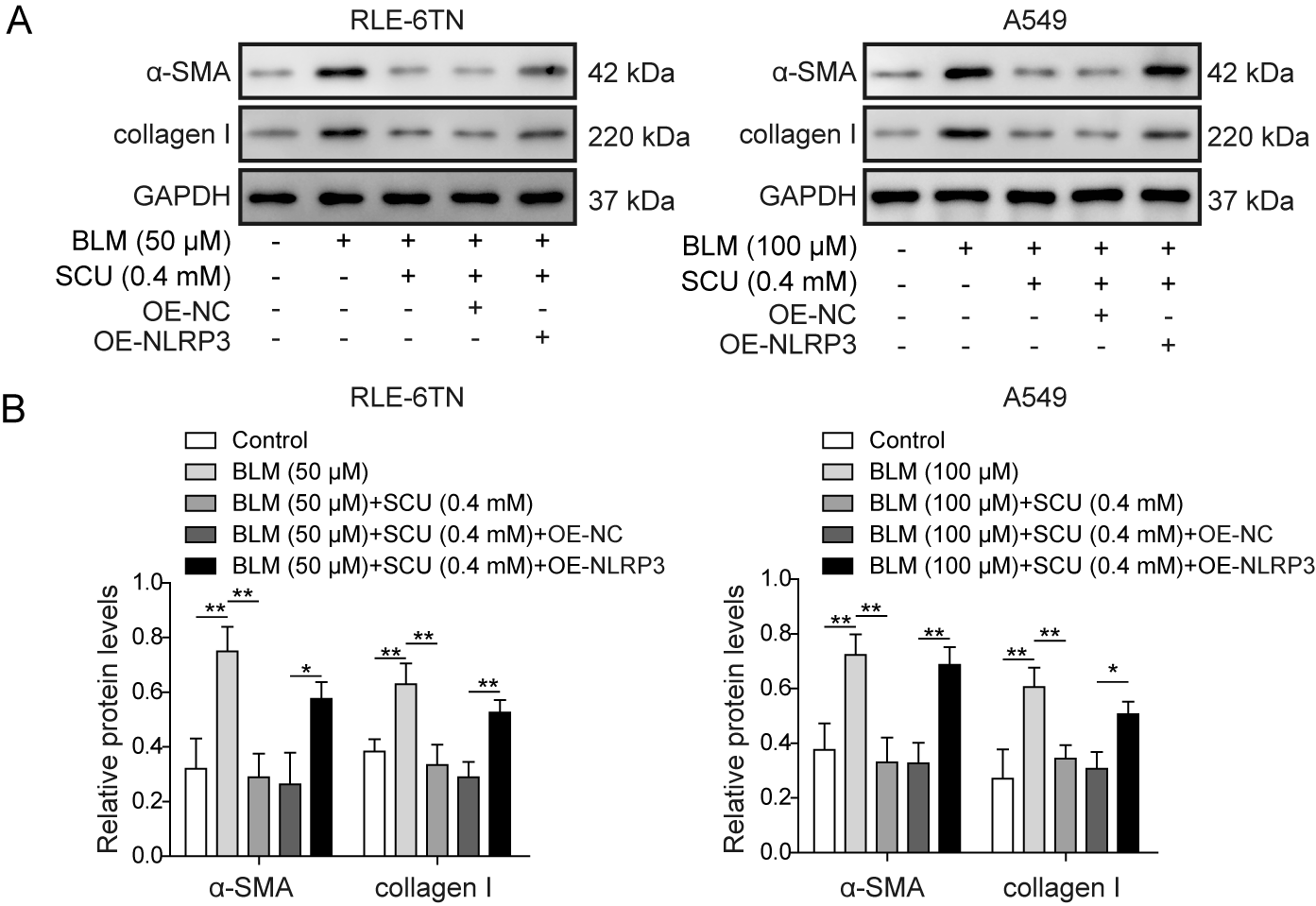

Supplement: Supplementary file 7 — supplementary fig.5 [file 41419_2020_3178_MOESM7_ESM.tif]
